# Cardiac‐specific succinate dehydrogenase deficiency in Barth syndrome

**DOI:** 10.15252/emmm.201505644

**Published:** 2015-12-23

**Authors:** Jan Dudek, I‐Fen Cheng, Arpita Chowdhury, Katharina Wozny, Martina Balleininger, Robert Reinhold, Silke Grunau, Sylvie Callegari, Karl Toischer, Ronald JA Wanders, Gerd Hasenfuß, Britta Brügger, Kaomei Guan, Peter Rehling

**Affiliations:** ^1^ Department of Cellular BiochemistryUniversity Medical Center GöttingenGöttingenGermany; ^2^ Department of Cardiology and PneumologyUniversity Medical Center GöttingenGöttingenGermany; ^3^ Heidelberg University Biochemistry CenterUniversity HeidelbergHeidelbergGermany; ^4^ German Center for Cardiovascular Research (DZHK)GöttingenGermany; ^5^ Departments of Clinical Chemistry and PediatricsAcademic Medical CenterUniversity of AmsterdamAmsterdamThe Netherlands; ^6^ Heart Research Center GöttingenGöttingenGermany; ^7^ Max Planck Institute for Biophysical ChemistryGöttingenGermany

**Keywords:** Barth syndrome, cardiolipin, mitochondria, respiratory chain, succinate dehydrogenase, Cardiovascular System, Genetics, Gene Therapy & Genetic Disease, Metabolism

## Abstract

Barth syndrome (BTHS) is a cardiomyopathy caused by the loss of tafazzin, a mitochondrial acyltransferase involved in the maturation of the glycerophospholipid cardiolipin. It has remained enigmatic as to why a systemic loss of cardiolipin leads to cardiomyopathy. Using a genetic ablation of tafazzin function in the BTHS mouse model, we identified severe structural changes in respiratory chain supercomplexes at a pre‐onset stage of the disease. This reorganization of supercomplexes was specific to cardiac tissue and could be recapitulated in cardiomyocytes derived from BTHS patients. Moreover, our analyses demonstrate a cardiac‐specific loss of succinate dehydrogenase (SDH), an enzyme linking the respiratory chain with the tricarboxylic acid cycle. As a similar defect of SDH is apparent in patient cell‐derived cardiomyocytes, we conclude that these defects represent a molecular basis for the cardiac pathology in Barth syndrome.

## Introduction

Barth syndrome (BTHS) is an X‐chromosome‐linked, inherited disease, which is associated with cardiomyopathy, along with skeletal myopathy, growth retardation, and neutropenia (Barth *et al*, 1983). The affected gene encodes for the mitochondrial protein tafazzin, an enzyme involved in the synthesis of the mitochondrial lipid, cardiolipin (CL) (Neuwald, 1997).

Cardiolipin is a dimeric glycerophospholipid with a glycerol head group and two phosphatidic acid backbone molecules. Tissues with high mitochondrial metabolic activity require a specific fatty acid composition within CL. In the case of the mammalian heart, four linoleic acid (18:2) molecules are bound (Hoch, 1992). The tissue‐specific fatty acid composition of cardiolipin is a result of a remodeling process after its initial synthesis in the inner membrane of mitochondria (Osman *et al*, 2011). CL is remodeled in a two‐step process: First, CL is deacylated to form monolysocardiolipin (MLCL), and then this is subsequently reacylated by the CoA‐independent phospholipid acyltransferase, tafazzin (Osman *et al*, 2011). As this remodeling maintains the physiological amount and species composition, a tafazzin defect in BTHS patients causes a decrease in mature CL species and an increase in their precursor, MLCL (Schlame *et al*, 2003). Changes in CL amount, species distribution, and peroxidation status have been found associated with several cardiac disorders, including failing and aging heart, as well as ischemia and reperfusion injury. In addition, CL alterations have also been implicated in the diabetic heart (Paradies *et al*, 2004; Han *et al*, 2005; He & Han, 2014; Mulligan *et al*, 2014).

The glycerophospholipid cardiolipin is almost exclusively localized within mitochondria and predominantly (75%) present in the inner mitochondrial membrane (Gebert *et al*, 2009). CL has been implicated in a variety of conserved mitochondrial functions. It is considered to be involved in maintaining mitochondrial morphology, including the formation of inner membrane cristae structures (Xu *et al*, 2005), as well as Fe‐S biogenesis, apoptosis, and mitochondrial metabolism (Guiseppe, 2014).

In cardiac tissue, 90% of the energy demand is provided by the oxidative phosphorylation system in the inner mitochondrial membrane. The respiratory chain consists of four complexes, I, II, III, and IV, which are involved in the transport of electrons from oxidized metabolites to the terminal acceptor, molecular oxygen. Electron transport is coupled to the export of protons, resulting in a membrane potential, which drives the synthesis of ATP by the F_1_F_o_‐ATP synthase (complex V). In recent years, ample evidence has been generated showing that individual respiratory chain complexes oligomerize in the inner membrane to form large supercomplexes, so‐called respirasomes. A close interaction of complex I with a dimer of complex III forms a platform to which several copies of complex IV are recruited (Schägger & Pfeiffer, 2000, 2001; Althoff *et al*, 2011). The formation of supercomplexes is thought to enhance the efficiency of energy transfer between complexes and to prevent the generation of reactive oxygen species (Acín‐Pérez *et al*, 2008; Chen *et al*, 2012; Strogolova *et al*, 2012; Vukotic *et al*, 2012). Succinate dehydrogenase (complex II) is not part of the supercomplexes, but forms a separate complex in the inner mitochondrial membrane. Succinate dehydrogenase consists of four subunits, of which subunits C and D form a membrane anchor. Subunit A is covalently bound to flavin and forms the substrate binding site. Subunit B is involved in the transfer of electrons to the ubiquinone pool. Cellular models of BTHS have been found useful in biochemical studies to establish a role for cardiolipin in stabilizing respiratory supercomplexes (Brandner *et al*, 2005; Xu *et al*, 2005; Mckenzie *et al*, 2006; Dudek *et al*, 2013; Gonzalvez *et al*, 2013). However, it remains unclear whether these models recapitulate the situation in cardiac tissue in the context of BTHS.

Here, we report on two complementary approaches to assess mitochondrial defects in heart tissue. For this, we utilized BTHS patient‐induced pluripotent stem cell (iPSC)‐derived cardiomyocytes and a BTHS mouse model, in which an inducible systemic knockdown of tafazzin gene (*TAZ*) expression causes a reduction in mature forms of CL (Acehan *et al*, 2011; Soustek *et al*, 2011). In initial analyses, the mouse model showed a reduction in cardiac left ventricle ejection fraction (EF), coinciding with the development of a cardiomyopathy phenotype, but only after eight months of continuous tafazzin knockdown (Acehan *et al*, 2011; Soustek *et al*, 2011). To assess mitochondrial malfunctions at the molecular level prior to the onset of a pathophysiology, we performed biochemical analyses of tissue samples from 2‐month‐old mice. At this age, the BTHS mice did not display cardiomyopathy despite significant alterations in cellular mitochondrial cardiolipin organization profiles. Our analyses show that mitochondria from cardiac tissue exhibit a severe reorganization of the respiratory chain and concomitantly a significant reduction in respiratory activity, whereas kidney and liver mitochondria are not affected at this stage. Interestingly, compared to liver and kidney, a striking selective reduction in succinate dehydrogenase activity was observed in cardiac tissue. This decrease in enzyme function is due to a tissue‐specific loss of active complex II. Using BTHS patient‐derived cardiomyocytes, we were able to confirm the remodeling of the respiratory chain and the deficiency in succinate dehydrogenase in humans. Based on these findings, we propose that alterations in respiratory chain organization and a lack of succinate dehydrogenase activity impact the metabolism of cardiac cells in BTHS.

## Results

### Cardiolipin profiling at a pre‐onset stage in the Barth syndrome mouse model

**Figure 1:**
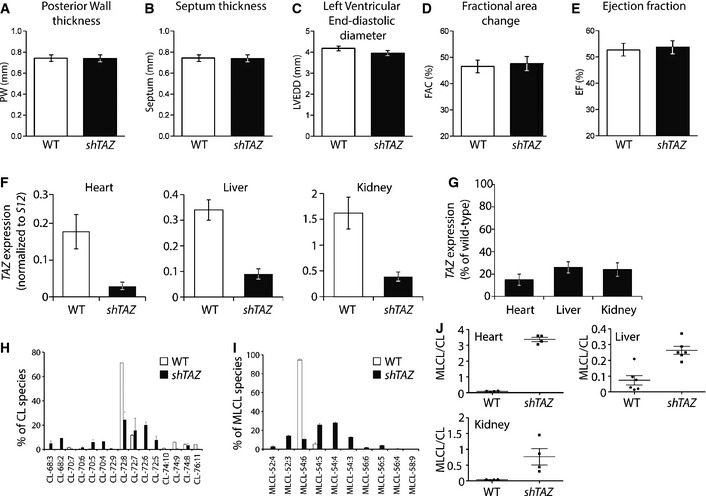
Cardiolipin species composition and effects on cardiac function in tafazzin‐deficient mice (*shTAZ*) compared to control (WT) A–EEchocardiographic analysis of (A) posterior wall thickness, (B) septum thickness, (C) left ventricular end‐diastolic diameter, (D) fractional area change, and (E) ejection fraction. Mean of *n *= 8 per genotype ± SEM.FComparison of *TAZ* gene expression, normalized to S12, in the heart, liver, and kidney, analyzed in triplicate by quantitative PCR. Mean of *n *= 5 per genotype ± SEM.G
*TAZ* gene expression in percent. Wild type is set to 100%; error bars represent SEM.H, ILipid species profiles of cardiolipin (H) and monolysocardiolipin (I) in isolated cardiac mitochondria analyzed by mass spectrometry. Mean of *n *= 4 per genotype ± SEM.JComparison of ratios of total monolysocardiolipin to cardiolipin content in indicated tissues.

In the BTHS mouse model (*ROSA26* ^*H1/tetO‐shRNA:TAZ*^), the administration of doxycycline systemically induces a short hairpin RNA, which reduces tafazzin gene expression (Acehan *et al*, 2011; Soustek *et al*, 2011). To study the changes in mitochondrial physiology before the onset of a cardiac phenotype, *ROSA26* ^*H1/tetO‐shRNA:TAZ*^ (*shTAZ*) and wild‐type mice were treated with doxycycline for 2 months and cardiac function was assessed. Echocardiographic analyses showed normal heart dimensions (posterior wall thickness and septum thickness) and normal left ventricular end‐diastolic diameter (LVEDD: WT vs. *shTAZ* 4.19 ± 0.07 mm vs. 3.96 ± 0.11 mm; Fig 1A–C). Analysis of heart function revealed no difference between *shTAZ* and WT mice in the fractional area change (FAC): WT vs. *shTAZ* 46 ± 1% vs. 47 ± 1%, or in the ejection fraction (EF): WT vs. *shTAZ* 53 ± 3% vs. 54 ± 1%, (Fig 1D and E). These data demonstrate that at this early stage, the BTHS mouse model showed no signs of cardiomyopathy.

**Figure EV1:**
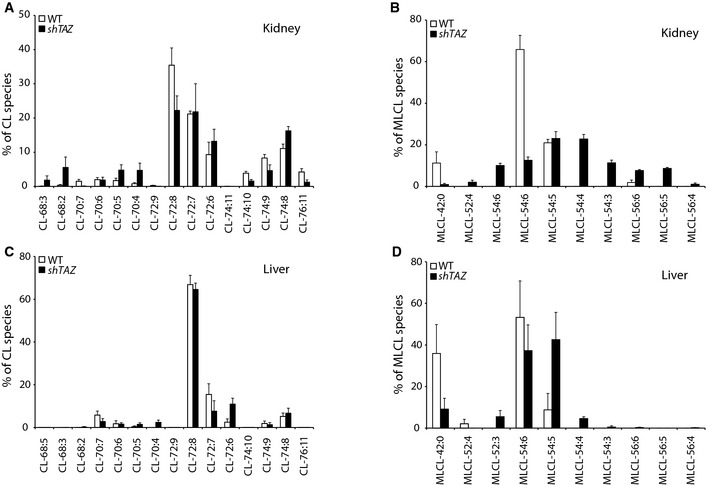
Cardiolipin and monolysocardiolipin profiles in kidney and liver A–DLipid species profiles of cardiolipin (A, C) and monolysocardiolipin (B, D) in the mitochondria isolated from indicated tissues analyzed by mass spectrometry. Mean of *n* = 4 per genotype, ± SEM.

The systemic down‐regulation of *TAZ* in the BTHS mouse model was validated by the analysis of *TAZ* mRNA levels using quantitative PCR in three different tissues: heart, liver, and kidney (Fig 1F). When the percentage of *TAZ* expression compared to wild type was calculated, all three tissues showed a similarly robust down‐regulation of *TAZ* gene expression (Fig 1G). Based on this observation, we measured the lipid profiles of the three selected tissues (heart, liver, and kidney) by mass spectrometry to verify that the *shTAZ* knockdown in the mouse model reflected a true cardiolipin deficiency. Cardiac tissue is unique in its cardiolipin distribution as the tetralinoleic acid CL (72:8) species is the most abundant form in this tissue, followed by CL (72:7), which represents a prominent species only in mouse (Minkler & Hoppel, 2010). Lipid extracts of mitochondria isolated from the hearts of BTHS mice showed a strong specific reduction in the tetralinoleic acid (72:8) species of cardiolipin compared to control mice (Fig 1H). Furthermore, the enzymatic block in CL biosynthesis caused an increase in total levels of its precursor MLCL. Normalization of MLCL species distribution to the total MLCL amount in *shTAZ* animals revealed a broad change in the distribution pattern (Fig 1I). In contrast to heart, kidney and liver tissues have a significantly broader species distribution and tetralinoleic CL (72:8) is less prominent (Fig EV1). Knockdown of *TAZ* gene expression, besides its effect on CL, also causes changes in the MLCL pool in both kidney and liver tissues, which were comparable to the changes in the heart. In agreement with an altered distribution of CL species in heart, kidney, and liver, a loss of tafazzin affects each of the various CL species to a different extent (Fig EV1). As expected, the MLCL/CL ratio was clearly elevated in *shTAZ* animals in all three organs (Fig 1J). In summary, despite the systemic knockdown of *TAZ* and the strong reduction in mature CL species in the heart, mice do not display signs of cardiomyopathy at the age of 2 months.

### Defects in respiratory performance in heart mitochondria from BTHS mice

**Figure 2:**
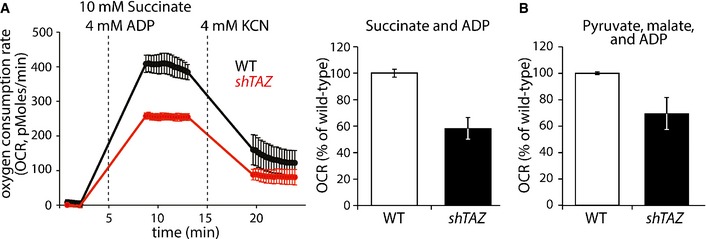
Reduced respiratory activity in cardiac mitochondria of *shTAZ* mice Periodic measurement of the oxygen consumption rate (OCR) of mitochondria, isolated from 5 mice per genotype, before and after the administration of the indicated compounds (left). Quantitation of OCR in independent experiments, *n* = 8 per genotype (right), mean ± SEM.OCRs of cardiac mitochondria after the administration of pyruvate, malate, and ADP, mean of *n* = 7 per genotype ± SEM.

Since mitochondria play a major role for cardiac energy metabolism, we first analyzed respiratory chain activity in the heart of *shTAZ* mice. Mitochondria isolated from the cardiac tissues of *shTAZ* and control mice were subjected to oxygen consumption analyses by real‐time respirometry using a Seahorse Extracellular Flux (XF) Analyzer. After the addition of the substrates succinate and ADP, a significantly reduced oxygen consumption rate (59%) was apparent in *shTAZ* mitochondria compared to the control (Fig 2A). Succinate promotes respiration by transferring electrons into the ubiquinone pool via complex II of the respiratory chain, thus bypassing complex I. Therefore, we also used pyruvate and malate as substrates. Oxidation of these substrates requires the activity of complex I. Under these conditions, respiration was decreased to 69% in cardiac *shTAZ* mitochondria compared to control mitochondria (Fig 2B). We conclude that despite the lack of a cardiac pathophysiology after 8 weeks of knockdown, mitochondria from *shTAZ* mice display substantial defects in respiratory chain function.

**Figure EV2:**
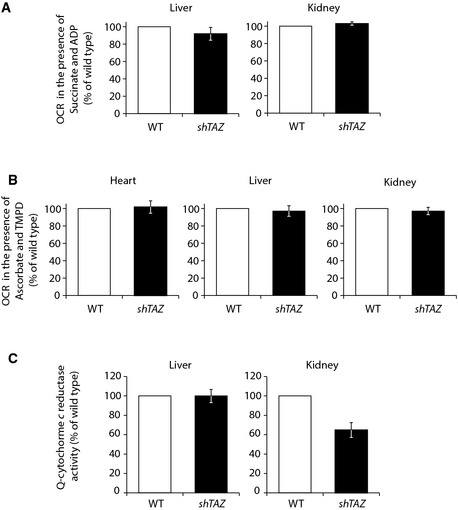
Respiration is not affected in kidney and liver Measurement of the oxygen consumption rate (OCR) of isolated mitochondria after the administration of succinate and ADP as substrate. Mitochondria from the liver (left) and kidney (right) were measured from 3 different animals per genotype, mean ± SEM.OCRs of mitochondria after the administration of TMPD and ascorbate, isolated from the heart (left; *n* = 8 per genotype), liver (central; *n* = 3 per genotype), and kidney (right; *n* = 3 per genotype); data represented in mean, ± SEM.Enzymatic activity of complex III in the liver (left; *n* = 3 per genotype) and kidney (right; *n* = 2 per genotype) normalized to MDH activity, in percent with control set to 100% (mean ± SEM). Complex III activity was measured spectrophotometrically as an increase in reduced cytochrome c over time.

The systemic knockdown of *TAZ* in the mouse model results in a cardiomyopathy, but defects in other organs have not been reported (Acehan *et al*, 2011; Soustek *et al*, 2011). As morphological changes in mitochondria have been reported in the liver of Barth syndrome patients (Bissler *et al*, 2002), we became interested whether respiration in mitochondria isolated from the liver and kidney of *shTAZ* mice is affected. Interestingly, succinate‐driven respiration was not impaired in mitochondria from *shTAZ* mice (Fig EV2A).

### Remodeling of the respiratory chain in cardiolipin‐deficient mitochondria in cardiac tissue

**Figure 3:**
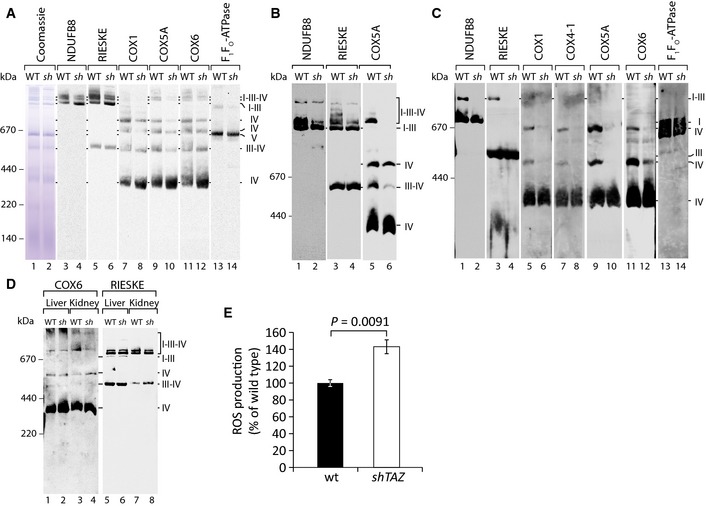
Structural rearrangement of the respiratory chain in tafazzin‐deficient mitochondria causes an increased generation of reactive oxygen species (ROS) Mitochondrial membranes, isolated from the cardiac tissue, were solubilized in 1% digitonin and analyzed by BN–PAGE and Western blotting with the indicated antibodies.Cardiac mitochondria were prepared as in (A) and analyzed on a large‐pore BN gel for better resolution of supercomplexes.Cardiac mitochondria were solubilized in 0.4% DDM, and the complexes were separated by BN–PAGE and analyzed by Western blotting with the indicated antibodies.Mitochondria were isolated from the kidney and liver and analyzed as in (A).Quantitation of ROS production over time in cardiac mitochondria using the ROS‐specific fluorescence‐based sensor H2DCFDA. One representative measurement is shown. Analyses were performed in triplicate and confirmed for three animals per genotype. Mean ± SEM, significance was calculated using Student's *t*‐test. *sh, shTAZ*.

Studies using tafazzin‐deficient cellular models indicate that cardiolipin is an important structural component of mitochondrial respiratory chain complexes and stabilizes their supercomplexes in the inner mitochondrial membrane (Xu *et al*, 2005; Mckenzie *et al*, 2006; Dudek *et al*, 2013; Gonzalvez *et al*, 2013). Hence, we used the mouse model to analyze the organization and amount of respiratory chain complexes in cardiac tissue. Mitochondria from the hearts of *shTAZ* and control mice were isolated and mitochondrial membrane protein complexes were solubilized in the mild detergent digitonin, which maintains intact respiratory chain supercomplexes. Respiratory chain complexes were then separated on blue native–polyacrylamide gels (BN–PAGE) and analyzed by Western blotting. Antibodies against complex I (NDUFB8) detected several forms of complex I‐containing supercomplexes in the high molecular weight range of the gel. These complexes represent complex I in association with complex III and with several copies of complex IV (Fig 3A). Antibodies against complex III (RIESKE) detected the supercomplexes as well as a smaller‐sized complex, comigrating with complex IV (III–IV). When using antibodies against complex IV (COX1, COX5A, COX6), three complexes with a lower molecular weight than the supercomplexes were visible. In addition, the prominent monomeric form of cytochrome c oxidase, at about 400 kDa, was present. In comparison with wild‐type mitochondria, the quantity of supercomplexes (I‐III‐IV) was noticeably reduced in *shTAZ* mitochondria. In contrast, lower molecular weight forms, particularly the monomeric form of the cytochrome c oxidase, were more abundant in *shTAZ* mitochondria and appeared to migrate slightly faster than in the wild‐type samples. However, compared to the respiratory chain protein complexes, the F_1_F_o_‐ATPase complex was not significantly affected in *shTAZ* mitochondria (Fig 3A).

To dissect supercomplexes with increased resolution, large‐pore BN gels were used to separate solubilized mitochondrial protein complexes. This analysis showed that the majority of respiratory chain supercomplexes were strongly decreased in *shTAZ* mitochondria (Fig 3B). The only large complex, which consistently remained stable in *shTAZ* mitochondria, was the complex I–III oligomer, comprising complex I and dimeric complex III (Fig 3A and B). In order to analyze whether the stability of the complex I–III oligomer was maintained under harsher detergent conditions, dodecyl maltoside (DDM) was used for solubilization. In a BN gel separation of DDM‐treated mitochondria, supercomplexes disintegrate into the monomeric form of complex I, the dimeric form of complex III, and low molecular weight forms of complex IV (Mckenzie *et al*, 2006). The oligomer of complexes I and III (I–III) remained the largest detectable complex in wild‐type mitochondria. However, under these conditions, this complex is selectively dissociated in *shTAZ* mitochondria (Fig 3C). The reduced complexity of the supercomplex pattern upon DDM solubilization revealed that the total amount of respiratory chain complexes was similar in both samples. In agreement with this, the steady‐state protein levels of all tested mitochondrial proteins in both preparations, as assessed by SDS–PAGE, were equal (data not shown).

Based on the striking phenotype of cardiac mitochondrial supercomplexes, we addressed whether a similar redistribution could be observed in other tissues. To test this, mitochondria were isolated from the liver and kidney and solubilized by digitonin and protein complexes were separated by BN–PAGE. As seen in cardiac tissue samples, antibodies against complexes III (RIESKE) and IV (COX6) revealed supercomplexes and complexes of lower molecular weight in the liver and kidney mitochondria samples. Compared to the dramatic changes observed in the cardiac tissues, the respiratory chain complexes from the kidney and liver of the *shTAZ* mouse remained almost unchanged (Fig 3D). Taken together, cardiac mitochondria from *shTAZ* mice display pronounced remodeling of the respiratory chain, with a shift from supercomplex to low molecular weight forms.

Previous studies indicated that dissociation of respiratory chain complexes in the absence of the oligomerization factor Rcf1 leads to an increased formation of reactive oxygen species (ROS) (Chen *et al*, 2012; Strogolova *et al*, 2012; Vukotic *et al*, 2012). Hence, we directly analyzed whether the structural changes in heart tissue affects the efficiency of electron transfer within the respiratory chain and concomitantly leads to an increase in ROS formation. To this end, we isolated the mitochondria from cardiac tissue from *shTAZ* and control mice and performed real‐time ROS measurements. Using the fluorescence‐based ROS sensor H2DCFDA, the short‐time kinetic approach revealed a significant increase in ROS within ten minutes of measurement (Fig 3E). In summary, cardiac mitochondria from BTHS mice display an increased dissociation of respiratory chain supercomplexes, consistent with an increase in the formation of reactive oxygen species.

### Mitochondria from BTHS mice display specific enzyme deficiencies

**Figure 4:**
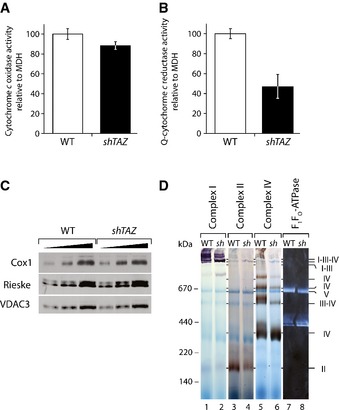
Cardiolipin deficiency differently affects individual respiratory chain complexes Enzymatic activity of ubiquinone‐cytochrome c oxidase in the mitochondria isolated from the cardiac tissues. The ubiquinone analogue decylubiquinone was used in this assay. Data were standardized to malate dehydrogenase activity and presented as percent of wild type, mean of *n* = 4 per genotype, ± SEM.Cytochrome c reductase activity, standardized to malate dehydrogenase activity in the cardiac mitochondria, was plotted as % of wild type, mean of *n* = 3 per genotype, ± SEM.Protein levels of Cox1 and Rieske compared to VDAC3 in three different dilutions of isolated mitochondria, separated on SDS‐PAGE, and visualized by Western blotting.Respiratory chain complexes in cardiac mitochondria were separated on BN–PAGE and stained for activity of complexes I, II, IV, and F_1_F_O_‐ATPase. *sh, shTAZ;* Q, ubiquinone.

While the dissociation of supercomplexes was in agreement with an increase in ROS production, this phenotype alone did not fully explain the observed reduced OCR of the BTHS mice. Hence, the enzymatic activities of individual complexes of the respiratory chain in solubilized cardiac mitochondria were analyzed. As a reference, the activity of malate dehydrogenase (MDH), a soluble enzyme of the tricarboxylic acid cycle, was measured. Complex IV activity, as determined by spectrometric analysis of cytochrome c oxidation, was not significantly affected in *shTAZ* mitochondria compared to wild type (Fig 4A). Cytochrome c oxidase (complex IV) is the terminal enzyme of the respiratory chain and reduces molecular oxygen to water. To confirm that this enzyme is not affected, the oxygen consumption rate was measured using the artificial electron donor TMPD (N,N,N',N'‐tetramethyl‐p‐phenylenediamine dihydrochloride) in the presence of ascorbate. While ascorbate retains TMPD in the reduced state, TMPD transfers electrons to cytochrome c and thus, via this electron carrier, electrons are fed directly into complex IV. This enables the bypassing of preceding complexes of the respiratory chain (Granger & Lehninger, 1982). In agreement with the above results, respiration was not affected in *shTAZ* mitochondria (Fig EV2B). Next, we addressed the enzymatic activity of complex III by monitoring the reduction of exogenously supplemented cytochrome c. These analyses revealed a clear decrease in complex III activity in *shTAZ* mitochondria (48% of wild type) (Fig 4B). Hence, we analyzed whether the reduced complex III activity was a consequence of decreased amounts of the complexes. After DDM solubilization of mitochondria, equal amounts of complex III were apparent (Fig 3C) and identical protein amounts of one of the core components, the Rieske iron sulfur protein, were detected in SDS‐PAGE separations (Fig 4C). These analyses indicate a specific loss of complex III activity due to cardiolipin deficiency in the cardiac tissue of the BTHS mouse model, thereby explaining the reduced respiration on succinate and pyruvate/malate observed previously (Fig 2A and B). Based on these findings, we assessed the enzymatic activity of complexes III and IV in the liver and kidney. Complex IV activity was determined by the measurement of the OCRs in the presence of TMPD/ascorbate and revealed no difference compared to control (Fig EV2B). Complex III activity was determined by reduction of externally added cytochrome c and normalized to MDH activity. While complex III shows no defect in liver, there was a slight reduction in its activity in the kidney, although this was not as pronounced as in cardiac tissue (Fig EV2C).

In a complementary approach, the activity of the individual respiratory chain complexes and supercomplexes was analyzed by in‐gel activity staining, followed by the separation of the complexes by BN–PAGE. This approach enabled the correlation of the activity of respiratory chain complexes with their position on the BN–PAGE gel. When individual enzymatic activities were visualized with specific staining methods (Fig. 4D), the resulting staining corresponded to the previously identified positions in the Western blot analyses (Fig 3A). Staining for complex I revealed a prominent activity in the range of supercomplexes in wild‐type mitochondria (indicated as I‐III‐IV in Fig 4D). The reduction of this supercomplex‐associated complex I activity in *shTAZ* mice corresponds well with the reduction of supercomplexes analyzed by Western blotting (Fig 3A and B). Complex IV‐specific staining was found in all forms previously identified by Western blot (labeled with IV, III–IV, and I‐III‐IV; Fig 3A). Interestingly, in BTHS mitochondria, almost the entire activity of complex IV was confined to the monomeric form. When complex II activity was assessed, a defined succinate dehydrogenase complex activity was detected in control mitochondria. Surprisingly, this activity was greatly reduced in the corresponding complex from *shTAZ* mitochondria (Fig 4D). Therefore, while supercomplex‐forming respiratory chain complexes display the expected reorganization in BTHS cardiac mitochondria, activity staining revealed a significant and unexpected loss of complex II activity.

### Succinate dehydrogenase deficiency in cardiac mitochondria of the BTHS mouse model

**Figure 5:**
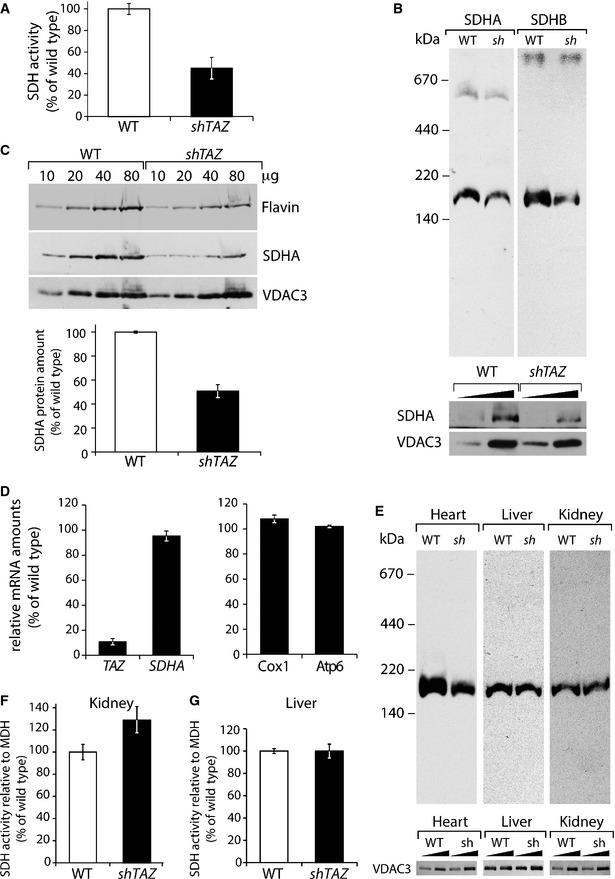
Structural and functional defects on the succinate dehydrogenase in the heart of *shTAZ* mice AEnzymatic activity of the succinate dehydrogenase in the isolated cardiac mitochondria from three animals per genotype, mean ± SEM.BMitochondrial membranes were solubilized in 1% digitonin and analyzed by BN–PAGE and Western blotting using antibodies against SDH components SDHA and SDHB (upper panel). For a loading control, samples were separated on SDS‐PAGE and analyzed by Western blotting (lower panel).CSteady‐state protein levels analyzed by SDS‐PAGE separation of indicated amounts of mitochondrial protein and subsequent Western blotting with antibodies against SDHA and VDAC3 or visualizing of flavin fluorescence after excitation with 488 nm. Lower panel: quantitation of signal ratio SDHA/VDAC3 in *shTAZ* and control (set to 100%), mean ± SEM.DAnalysis of gene expression in cardiac tissue analyzed in (C) by qPCR using primers against indicated mRNA. Data of one representative measurement, normalized to β‐actin with WT set to 100%, are shown, mean ± SEM. Identical results were obtained in further experiments with two different pairs of animals.EUpper panel: SDH complex of cardiac, liver, and kidney was analyzed by BN–PAGE as in (B); lower panel: the same samples were analyzed by SDS‐PAGE and Western blotting.F, GAnalysis of enzymatic activity of the succinate dehydrogenase related to the malate dehydrogenase activity in the mitochondria isolated from the kidney (F) and liver (G). Values of three animals per genotype are plotted as percent of wild type, mean ± SEM. *sh, shTAZ*.

Since a decrease in complex II activity has not been described in the context of Barth syndrome, we decided to further investigate this. As activity staining is not a fully quantitative approach, enzyme activity measurements were performed using purified mitochondria from the wild type and *shTAZ* cardiac tissue. In agreement with the in‐gel activity assay, complex II activity was drastically reduced to app. 48% in *shTAZ* cardiac mitochondria compared with wild‐type mitochondria (Fig 5A). The observed reduced succinate dehydrogenase (SDH) activity could be explained either by a defect in enzyme function or by an overall reduction of the complex. To discriminate between these possibilities, the succinate dehydrogenase complex was analyzed by BN–PAGE using antibodies against the A and the B subunits. In cardiac *shTAZ* mitochondria, the amount of succinate dehydrogenase was clearly reduced for both subunits (Fig 5B). In order to address whether this SDH complex reduction results from reduced amounts of individual SDH complex subunits, protein amounts of SDHA were analyzed by SDS–PAGE and Western blotting. Compared to the mitochondrial outer membrane protein VDAC3, the steady‐state level of SDHA was drastically reduced in the cardiac mitochondria from *shTAZ* mice (Fig 5C). The flavin group, which is covalently bound to the subunit A, allows a quantitative determination of SDHA by the detection of its autofluorescence in the SDS‐PAGE and revealed a similar decrease in quantity (Fig 5C). We next asked whether the reduced protein amount was the result of reduced gene expression. Therefore, levels of *SDHA* transcripts in the cardiac tissue were measured by quantitative PCR and, compared to the control, *SDHA* was expressed to a similar extent in *shTAZ* cardiac tissue (Fig 5D). To correlate this result with *TAZ* expression, the expression of *TAZ* in control and knockdown cells was assessed. The levels of *TAZ* mRNA were reduced to 10% in the *shTAZ* sample, whereas COX1 and ATP6 transcripts were comparable in both tissues.

The observed loss of succinate dehydrogenase in the cardiac mitochondria was especially interesting considering that fatty acids are the main supply of energy for myocardial contraction. Thus, the effect of cardiolipin deficiency on the SDH complex in the kidney and liver was also tested. Mitochondria from both tissues were analyzed by BN–PAGE using antibodies against SDHA. In contrast to the strong reduction of complex II in *shTAZ* cardiac mitochondria, the levels of this complex were not affected in the liver and kidney (Fig 5E). A direct measurement of SDH enzyme activity in the liver and kidney mitochondria demonstrated that complex II activity was not affected in the liver and even mildly increased in the kidney mitochondria of *shTAZ* animals (Fig 5F and G). Thus, we conclude that a loss of tafazzin function leads to a selective succinate dehydrogenase deficiency in the cardiac tissue.

### BTHS iPSC‐derived cardiomyocytes exhibit irregular sarcomeric organization but show no difference in cell size

**Figure 6:**
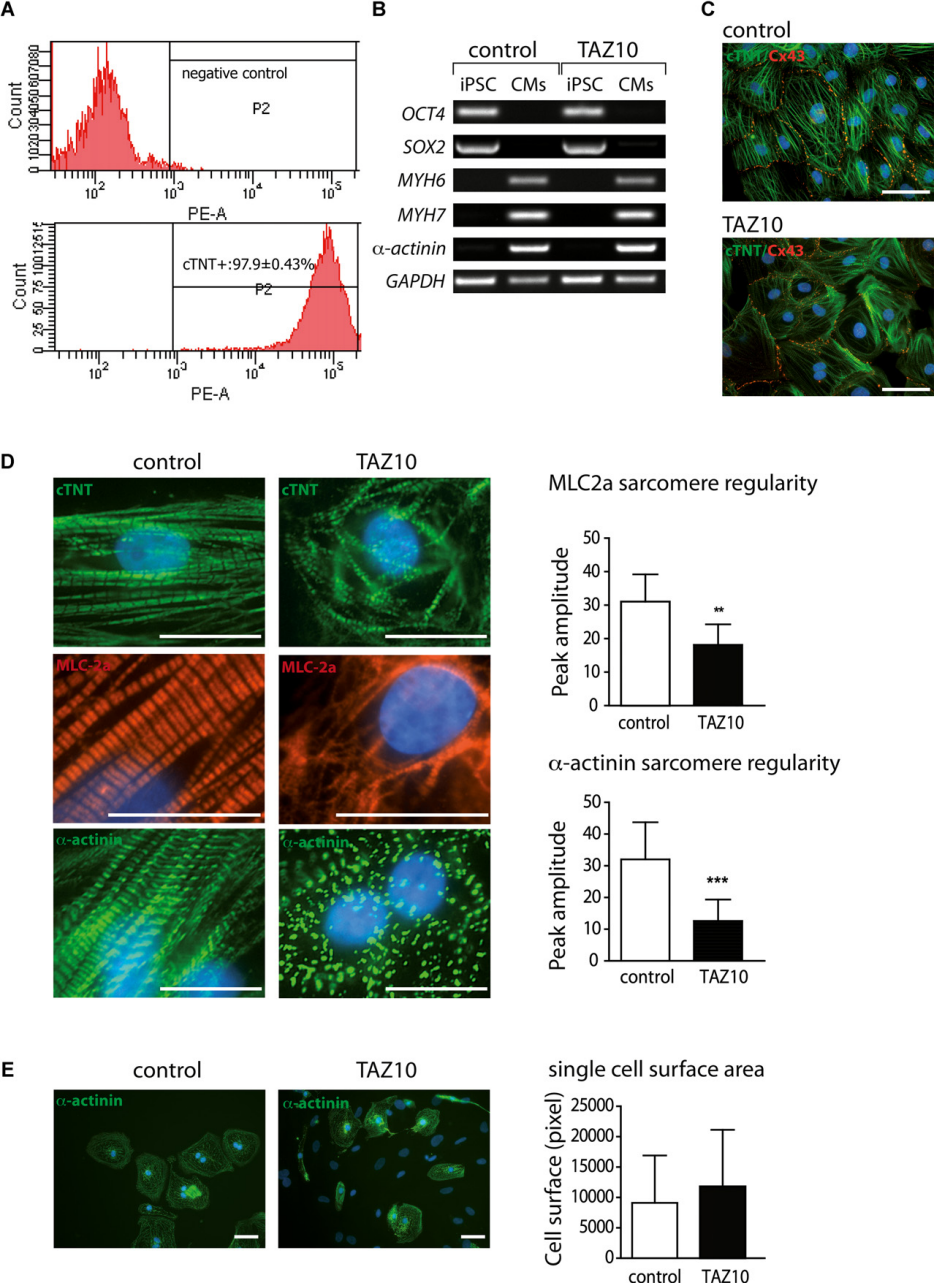
Irregularities in the sarcomeric structure in patient iPSC‐derived cardiomyocytes Flow cytometry analysis of cardiac troponin T (cTNT) expression was performed at day 60 post‐cardiac differentiation; 98% of cTNT^+^ cardiomyocytes (CM) were obtained after metabolic selection.RT‐PCR showing the expression of indicated genes in undifferentiated iPSCs and differentiated iPSC‐CMs of BTHS patient TAZ10 and a healthy control.Immunostaining of control (left) and BTHS iPSC‐CMs at day 60 post‐differentiation for cardiac troponin T (cTNT) and connexin 43 (Cx43). Scale bars: 50 μm.Quantitation of sarcomere organization. Left panel: Immunostaining of cTNT, MLC (myosin light chain) 2a, and α‐actinin at day 60 post‐cardiac differentiation. Right panel: The bar graphs represent the sarcomere regularity from three independent experiments. Error bars represent standard deviation for the comparison between control and BTHS patient TAZ10. Significance was analyzed using Student's *t*‐test, **P = 0.002 and ***P = 0.0006. Scale bars: 50 μm.Measurement of cell surface areas. Cell surface area was measured in single cardiomyocytes (*n* = at least 30 measurements each group from *N* = 3 independent experiments each group, mean ± SEM) at day 60 post‐cardiac differentiation after immunostaining of sarcomeric α‐actinin. Scale bars: 50 μm.

The findings of a cardiac‐specific pathology in tafazzin‐deficient mice triggered our interest in the pathogenesis of human cardiomyocytes. In our previous studies, we were able to generate iPSCs from the fibroblasts of BTHS patients (Dudek *et al*, 2013). Both control and BTHS iPSCs were differentiated into cardiomyocytes using the standardized protocol described previously (Lian *et al*, 2013) in combination with the metabolic purification of cardiomyocytes (Tohyama *et al*, 2013). At day 10, both control and BTHS iPSCs were differentiated into sheets of cardiomyocytes that beat spontaneously (Videos EV1 and EV2). After metabolic selection, cardiomyocytes were replated as monolayers (Videos EV3 and EV4) and cultured until day 60. Flow cytometry data showed that about 98% of cells at day 60 were positive for cardiac troponin T (cTNT; Fig 6A). No significant differences were observed between control and BTHS iPSCs regarding their differentiation efficiency. The cardiomyocyte cultures derived from both control and BTHS iPSCs expressed high levels of cardiac‐specific genes, such as *MYH6*,* MYH7,* and *α‐actinin*, whereas the expression of pluripotency markers, *OCT4* and *SOX2*, was significantly decreased (Fig 6B). No obvious differences were observed between control and BTHS iPSC‐derived cardiomyocytes.

Furthermore, double immunofluorescence staining revealed that both control and BTHS cardiomyocytes expressed the gap junction protein connexin (Cx43) at cell‐to‐cell contact sites in cardiac monolayers (Fig 6C), indicative of cell‐to‐cell communication. Following staining with antibodies against cTNT (Fig 6C and D), myosin light chain 2a (MLC2a; Fig 6D), and sarcomeric α‐actinin (Fig 6D), sarcomeric striations were found in cardiomyocytes derived from both control and BTHS iPSCs. However, sarcomere assembly in BTHS iPSC‐derived cardiomyocytes appeared more irregular than in control iPSC‐derived cardiomyocytes (Fig 6D). In control cells, the regular filament alignment was observed and the expression of cTNT, α‐actinin, and MLC‐2a was at evenly spaced intervals. In TAZ10 cells, the majority of cells showed punctate cTNT and α‐actinin distribution, as well as disturbed myosin filament assembly (Fig 6D).

To quantitate the regularity of sarcomere organization, two‐dimensional fast Fourier transform (2D FFT) of the immunostaining was used, for α‐actinin and MLC2a, to analyze the regularity of sarcomere spacing in control and BTHS iPSC‐derived cardiomyocytes. α‐Actinin, a cardiac Z‐disk major component, and MLC‐2a were used as markers to assess the sarcomeric Z‐disk and myosin filament assembly, respectively. The striation‐associated periodic signal amplitude of the first‐order peaks for both MLC2a and α‐actinin was significantly lower in BTHS cardiomyocytes in comparison with the control cells (Fig 6D). This indicates the irregularity of sarcomere organization or myofilament structure in BTHS cardiomyocytes. Notably, when cell surface size was measured, no significant difference was observed between BTHS iPSC‐derived cardiomyocytes and control cells (Fig 6E). These data suggest that BTHS iPSC‐derived cardiomyocytes could recapitulate disease phenotypes such as disturbed sarcomere organization with normal cell size, as previously described in the BTHS mouse model (Acehan *et al*, 2011; Soustek *et al*, 2011).

### Impaired respiration in cardiomyocytes derived from BTHS patient stem iPSCs

**Figure 7:**
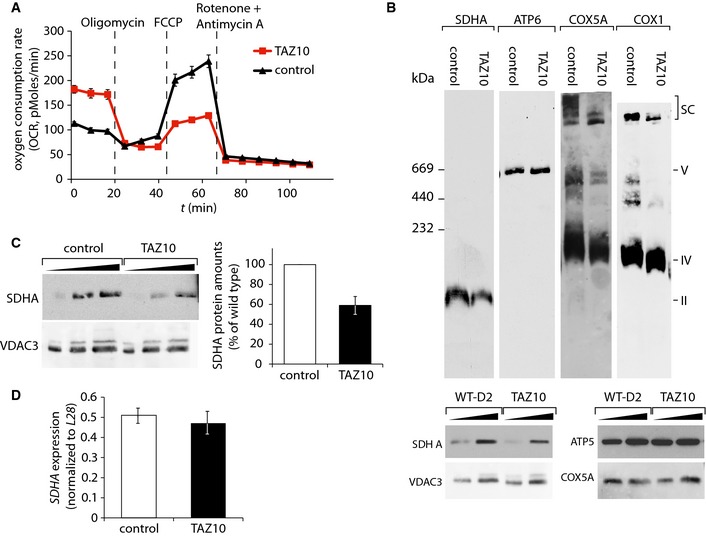
Succinate dehydrogenase deficiency in BTHS cardiomyocytes Oxygen consumption rate (OCR) of cardiomyocytes from patient TAZ10 and control were measured in eight technical replicates at basal conditions and after the administration of the indicated compounds, mean ± SEM.Mitochondrial membranes analyzed by BN–PAGE and Western blotting using indicated antibodies. For a loading control, samples were separated on SDS‐PAGE and analyzed by Western blotting (lower panel).Western blot analysis of SDHA protein levels compared to VDAC3. Quantitation of SDHA relative to VDAC3 in percent with control set to 100%. Error bars indicate range of values of two independent experiments.Analysis of *SDHA* gene expression in triplicate (mean ± SEM) by qPCR in cardiomyocytes analyzed in (C).

We addressed whether remodeling of the respiratory chain and defects in succinate dehydrogenase could also be observed in BTHS iPSC‐derived cardiomyocytes. To this end, oxygen consumption was measured using real‐time respirometry. Basal respiration in patient cells was significantly increased compared to cardiomyocytes from a healthy control, probably due to compensatory mechanisms, in agreement with published data (Wang *et al*, 2014). Determination of total respiratory capacity by FCCP uncoupling demonstrated a severe reduction in total capacity in BTHS cardiomyocytes, compared to the control (Fig 7A). Based on these observations, we analyzed whether a similar structural remodeling of the respiratory chain, as seen in mouse heart mitochondria, was apparent in the mitochondria from human cardiomyocytes. The structure of the respiratory chain complexes in the isolated mitochondria was analyzed by BN–PAGE, followed by Western blotting. Antibodies against COX5A and COX1 revealed a reduction of supercomplexes in the high molecular weight range in BTHS patient iPSC‐derived cardiomyocytes. Similar to cardiac mitochondria from BTHS mice, the majority of complex IV in BTHS iPSC‐derived cardiomyocytes resides in the monomeric form, which exhibits a slight shift in size (Fig 7B). Furthermore, the succinate dehydrogenase complex, analyzed using an antibody against the subunit SDHA, displayed a severe reduction in BTHS iPSC‐derived cardiomyocyte cells. In contrast, the F_1_F_o_‐ATPase did not display structural changes (Fig 7B). This observation led to the analysis of protein levels by Western blotting, using SDHA as a representative subunit of the complex. A reduction in SDHA levels to 59% was evident in BTHS patient‐derived cardiomyocytes (Fig 7C). Analysis of *SDHA* gene expression relative to *L28* by quantitative PCR revealed no difference in mRNA levels compared to control, suggesting instead an increased protein turnover in BTHS patient cardiomyocytes (Fig 7D). In summary, these data confirm that a structural remodeling of the respiratory chain complexes occurs in patient‐derived cardiomyocytes. Moreover, our analyses demonstrate that a defect in the succinate dehydrogenase can be recapitulated in human cardiac tissue.

## Discussion

The mitochondrial respiratory chain provides 90% of cardiac energy. Under physiological conditions, fatty acids serve as the main carbon source for cardiomyocytes (Grynberg & Demaison, 1996). The high β‐oxidation capacity of heart tissue is a reason for its elevated oxygen demand compared to other tissues. To cope with this high energy demand and the metabolic state, respiratory chain complexes are organized into large supercomplexes consisting of complex I, the dimeric *bc*_1_ complex, and cytochrome c oxidase (Schägger & Pfeiffer, 2000; Althoff *et al*, 2011). Ample evidence exists that cardiolipin is an integral part of individual respiratory chain complexes and is required for their enzymatic activity. Importantly, cardiolipin also participates in supercomplex function and stability (Eble *et al*, 1990; Sharpley *et al*, 2006; Bazán *et al*, 2012; Mileykovskaya *et al*, 2012). In the BTHS mouse model, we observed a reduction in complex III activity, whereas complex IV activity was not affected. This finding agrees with previous observations (Kiebish *et al*, 2013). Interestingly, we reveal that this molecular phenotype is apparent prior to the onset of a cardiac malfunction, suggesting that it is not a result of the pathological development, but rather causative for the development of the disease. Our data were assessed in mice aged 2 months, at which stage no cardiac phenotype was observed, consistent with previous results describing the development of cardiomyopathy only after 8 months of continuous knockdown (Acehan *et al*, 2011; Soustek *et al*, 2011).

Barth syndrome primarily affects cardiac function; however, it has remained unknown how a systemic defect in mitochondrial lipid composition leads to a tissue‐specific defect in metazoa. Here, we assessed the mitochondrial function in different tissues derived from the BTHS mouse model. Our analyses demonstrate a dramatic decrease in respiratory chain supercomplexes in the cardiac mitochondria of the BTHS mouse model. Similar changes were also apparent in human cardiomyocytes derived from BTHS patient iPSC. These structural alterations were not due to altered protein amounts as the steady‐state protein levels of respiratory chain subunits and complexes were not changed. In contrast to the defects in the heart, samples from the liver and kidney showed no significant alterations in the organization of respiratory chain complexes. The respiratory chain remodeling seen in the cardiac mitochondria is in agreement with the observations made in cellular BTHS model systems, such as iPSCs derived from patient fibroblasts (Dudek *et al*, 2013) and in Barth patient lymphoblasts (Mckenzie *et al*, 2006; Gonzalvez *et al*, 2013). However, the lack of a supercomplex dissociation phenotype in non‐cardiac tissue in the mouse model indicates that the situation in metazoan organisms differs significantly from that observed in the cellular models.

A direct comparison of the impact of tafazzin deficiency on the lipid pool in the heart, liver, and kidney shows that CL species distribution is fundamentally different between these tissues. Heart mitochondria appear characterized by the predominant presence of tetra‐linoleoyl‐CL (CL 72:8). We show that down‐regulation of *TAZ* dramatically changes MLCL species distribution in all tissues; however, individual species are affected differently in each tissue. It is unknown which CL species support respiratory chain complex structure and function and whether the accumulation of certain MLCL species might also have an effect on the structure of the respiratory chain.

It can only be speculated as to why cardiac is more susceptible to cardiolipin deficiency than other tissues. The requirement of cardiac tissue for one defined form of cardiolipin might be explained by cardiac‐specific isoforms of respiratory chain complex subunits (Grossman & Lomax, 1997). In complex IV, structural subunits that were found in close proximity to CL in the crystal structure dissociate under conditions of reduced CL, demonstrating a role in the structural integrity of the complex (Sedlák & Robinson, 1999). Interestingly, one of these CL‐dependent subunits (COXVIa) is expressed as a cardiac‐specific isoform and has an important regulatory function, thereby providing a possible explanation for the cardiac phenotype in BTHS patients.

Supercomplex formation is considered to increase the efficiency of the respiratory chain and to counteract the formation of reactive oxygen species (ROS) (Acín‐Pérez *et al*, 2008). In fact, in agreement with this concept, our analyses demonstrate that the formation of reactive oxygen species is significantly increased and already apparent in real‐time analyses. As ROS damage accumulates over the lifetime of an animal, we speculate that ROS contributes to the development of pathological symptoms after 8 months. As mitochondrial diseases are often correlated with an increased ROS production, targeting antioxidants into mitochondria has become a promising approach to reduce ROS levels and to prevent mitochondrial dysfunction. One of the most commonly used strategies is MitoQ. It is interesting to note that MitoQ treatment was recently shown to increase CL levels in the rat liver in addition to lowering ROS production (Fouret *et al*, 2015).

What are the physiological implications of supercomplex dissociation in the heart? Supercomplexes represent the active state of the mitochondrial respiratory chain. Consistent with this, we find that in the cardiac mitochondria of *shTAZ* mice, a strong reduction in the consumption of molecular oxygen over time is apparent. In addition, we found a decrease in the specific enzymatic activity of complex III, without an apparent reduction of its complex constituents or amounts. Structural analysis identified a CL binding site in close proximity to the ubiquinone binding site in complex III (Q_i_) indicative of a role for CL in the ubiquinone redox cycle (Wenz *et al*, 2009). This agrees with the observed reduced activity observed in *shTAZ* mice. Accordingly, the observed reduction in complex III activity explains the reduced respiration on pyruvate/malate and on succinate of the mitochondria isolated from the BTHS mouse model.

Interestingly, our analyses revealed a second cardiac‐specific defect. In the cardiac mitochondria of BTHS mice, the amount of complex II was severely reduced. The same defect, a reduction in SDH, was also found in the human cardiomyocytes derived from a BTHS patient iPSCs. In contrast, SDH levels were unaffected in the corresponding patient iPSCs (data not shown). Evidence for CL involvement in the formation of complex II in mitochondria is missing. However, a recent study showed that the reconstitution of this complex into nanodisks was only successful in the presence of CL (Schwall *et al*, 2012). Our data are in line with a role for CL in the structural integrity of complex II, as the active complex is severely reduced in *TAZ*‐knockdown mice, while the expression levels of the tested SDH subunits remained unchanged. Intriguingly, a defect of succinate dehydrogenase in the context of dilated cardiomyopathy in humans has been described previously (Davili *et al*, 2007). In this case, BTHS was ruled out since the patient did not display signs of neutropenia or aciduria; however, a lipid analysis has not been performed. Given the importance of β‐oxidation and the TCA cycle for the metabolism of the heart, we conclude that the succinate dehydrogenase deficiency observed in BTHS cardiac tissue likely contributes to cardiac malfunction. It is important to note that among the cellular models that we tested (*taz1*Δ yeast mitochondria, patient‐derived iPSCs), none displayed a complex II deficiency. While the dissociation of respiratory chain complexes has also been seen in cellular model systems, complex II deficiency was solely observed in the cardiac tissue. In some muscular disorders such as sarcopenia, an age‐dependent loss of muscle mass and reduction in complex IV activity correlate with an increase in complex II activity (Wanagat *et al*, 2001). Upregulation of SDH activity may represent a compensatory mechanism. It can be speculated that in cardiolipin‐deficient cardiac tissue, the requirements of SDH for CL may counteract this compensatory mechanism and therefore contribute to the susceptibility of cardiac tissue for CL deficiency.

In summary, here we show that in the BTHS mouse model, structural defects of the respiratory chain and a succinate dehydrogenase deficiency are restricted to cardiac tissue. We suggest that these early occurring defects play a fundamental role for the development of the cardiac defect in Barth syndrome.

## Materials and Methods

### Doxycycline transgene induction in BTHS mouse model

All animal procedures were performed in accordance with the German Animal Welfare Act and approved by the Landesamt für Verbraucherschutz und Lebensmittelsicherheit, Niedersachsen, Germany. Doxycycline (625 mg/kg) was administered as part of the standard rodent chow to wild‐type C57BL/6J mice and transgenic (*ROSA26* ^*H1/tetO‐shRNA:TAZ*^) animals. For breeding, female mice were treated with doxycycline 1 week before mating and doxycycline was withdrawn during mating to avoid male infertility. Doxycycline treatment was continued after detecting copulatory plugs, and pubs were continuously treated until 2 months of age. The genotype of the pubs was assessed by PCR, as described previously (Acehan *et al*, 2011).

### Echocardiography

Transthoracic echocardiography was performed blinded using a Vevo2100 (VisualSonics, Toronto, Canada) system with a 30‐MHz center frequency transducer. Briefly, animals were anesthetized with 3% isoflurane, and temperature‐, respiration‐, and ECG‐controlled anesthesia was maintained with 1.5% isoflurane. Two‐dimensional cine loops with frame rates of > 200 frames/s of a long‐axis view and a short‐axis view at mid‐level of the papillary muscles as well as M‐mode loops of the short‐axis view were recorded. Thicknesses of the septum, the posterior myocardial wall, the inner diameter of the left ventricle (LVEDD), and the area of the left ventricular cavity (area) were measured in systole (s) and diastole (d) from the short‐axis view according to the standard procedures (Collins *et al*, 2003). Maximal left ventricular length (L) was measured from the long‐axis view. Systolic and diastolic left ventricular volumes were calculated using the area–length method, and the ejection fraction (EF) was calculated out of the volumes. Measurements were obtained by an examiner blinded to the genotype of the animals.

### Cultivation and cardiac differentiation of patient‐specific iPSCs

All patient‐specific iPSCs (WT‐D2 and TAZ10) used in this study were generated and described previously (Dudek *et al*, 2013; Streckfuss‐Bömeke *et al*, 2013). The control iPSC line WT‐D2 was derived from a healthy individual, whereas TAZ10 was generated from a BTHS patients carrying the mutation c.590 G > T in the *TAZ* gene (Dudek *et al*, 2013). Feeder‐free adherent cultures of iPSCs were adapted from the feeder‐dependent cultures (Streckfuss‐Bömeke *et al*, 2013) by cultivating the iPSCs on Geltrex‐coated cell culture dishes in the presence of chemically defined medium E8 (Life Technologies). The cells were passaged every 4–5 days by using the non‐enzymatic Versene solution (Life Technologies). Differentiation of iPSCs into cardiomyocytes was performed by following the protocol as described previously (Lian *et al*, 2013). Briefly, undifferentiated iPSCs in feeder‐free system were cultured until confluent. To induce cardiac differentiation, the medium was replaced with RPMI + B27‐insulin medium, which contained RPMI 1640 medium (Life Technologies) supplemented with 2 mM l‐glutamine and B27 without insulin (Life Technologies). The GSK3 inhibitor CHIR99021 (10 μM, Millipore) was added to the culture for 24 h. Subsequently, the Wnt signaling inhibitor IWP2 (5 μM, Millipore) was added at day 3 for 2 days. At day 7, the culture medium was changed to RPMI + B27 + insulin, which contained RPMI 1640 medium supplemented with 2 mM l‐glutamine and B27 with insulin (Life Technologies), and culture medium was refreshed every 2–3 days. At day 20, the cells were first digested into single cells with collagenase IV (200 U/ml, Worthington) for 30 min and then with 0.25% trypsin/EDTA (Life Technologies) for 5 min and replated as monolayers into Geltrex‐coated culture dishes. Enrichment for cardiomyocytes was performed by adding 4 mM lactate in substitution of glucose for 6 days between days 20 and 30 (Tohyama *et al*, 2013).

### Flow cytometry

Enriched iPSC‐derived cardiomyocyte cultures at day 60 were dissociated with 0.25% trypsin/EDTA (Life Technologies) into single cells, fixed with 4% paraformaldehyde at room temperature for 20 min, and blocked with 2% bovine serum albumin (BSA) in phosphate‐buffered saline (PBS) for 1 h. Subsequently, cells were permeabilized with 0.1% Triton X‐100 (Sigma‐Aldrich) for 10 min at room temperature and then incubated with mouse monoclonal IgG antibody against cardiac troponin T (cTNT, Thermo Scientific) for 1 h at room temperature. Cells were then stained with PE donkey anti‐mouse IgG secondary antibody for 1 h at room temperature and subsequently analyzed using flow cytometer (BD FACS Aria II and FACSDiva software, BD Bioscience).

### Immunostaining

For marker expression, the measurement of cell size, and subcellular distribution, both control and BTHS iPSC‐derived cardiomyocytes at day 60 were fixed with 4% paraformaldehyde and blocked with 2% BSA in PBS. The following primary antibodies were used: mouse monoclonal IgG anti‐cTNT, mouse monoclonal IgG anti‐sarcomeric α‐actinin (Sigma‐Aldrich), rabbit polyclonal IgG anti‐connexin 43 (Cx43, Abcam), and mouse monoclonal IgG anti‐myosin light chain 2a (MLC2a, Synaptic Systems) antibodies. The cells were incubated for 1 h at room temperature with the primary antibody diluted in PBS with 1% BSA and then rinsed three times for 5 min with PBS. Afterward*,* cells were incubated with secondary antibodies: FITC goat anti‐mouse IgG (1:200) for cTNT and α‐actinin, Cy3 goat anti‐rabbit IgG (1:300) for Cx43, and Cy3 goat anti‐mouse IgG+IgM (1:300) for MLC‐2a. The cell nuclei were visualized with DAPI (4′,6‐diamidino‐2‐phenylindole, Sigma‐Aldrich) and examined by a fluorescence microscope (Zeiss Observer Z1).

### Measurement of cell size and sarcomere organization

For cell size measurements, images were made of α‐actinin‐stained cells using a fluorescence microscope (Zeiss Observer Z1) and analyzed using Image‐Pro plus image analysis software (Media Cybernetics). Microscope focus was optimized for the distinction of cell boundaries. To minimize the variability caused by the cell density, only single cardiomyocytes were chosen and outlined manually, and their surface areas were calculated automatically.

For a quantitative analysis of sarcomere organization, the images of immunostainings for α‐actinin and MLC2a were transferred to the ImageJ software (National Institutes of Health, Bethesda, MD). To get proper sarcomere organization measurements, the striation pattern of the α‐actinin and MLC2a staining was first aligned vertically within the field of view. The sarcomere organization was quantitated from a user‐defined region of interest (ROI) with at least 15 sarcomeres. A two‐dimensional fast Fourier transform (2D FFT) was processed on the selected ROI using the ImageJ software. The 2D FFT transforms the intensity trace from the spatial domain to the frequency domain. One‐dimensional representation of the 2D FFT is calculated by summing up the radial profiles of the 2D FFT, resulting in a series of peaks localized at integer multiples of the spatial frequency of the sarcomeric pattern. The amplitude of the first‐order peak is a measure of sarcomere organization, as the organization increases as more sarcomeric α‐actinin‐ or ML2a‐positive elements are localized regularly at a distance of the sarcomere length (Weiwad *et al*, 2000; Wagner *et al*, 2012).

Data are presented as mean ± standard deviation (SD). Significance was determined by Student's *t*‐test. A *P*‐value < 0.05 was considered statistically significant. The number of independent experiments and *P*‐values are indicated in the figure legends.

### Analysis of lipid profiles by mass spectrometry

Lipid extractions were performed as described previously (Ozbalci *et al*, 2013). Briefly, 7.5 μg protein was subjected to an acidic Bligh and Dyer extraction in the presence of 100 pmol PC (13:0/13:0, 14:0/14:0, 20:0/20:0; 21:0/21:0), 50 pmol CL (14:0/14:0/14:0/14:0, 14:1/14:1/14:1/15:1) and 25 pmol MLCL (16:0/16:0/16:0). Lipid standards were purchased from Avanti Polar Lipids. Evaporated lipid extracts were resuspended in 120 μl methanol/chloroform (2:1). Prior to measurement, samples were diluted 1:2 with 0.05% triethylamine in methanol. Mass spectrometric analysis of lipids was performed in negative ion mode on a Q‐Exactive from Thermo Scientific. Samples were automatically injected via a TriVersa NanoMate device (Advion). Spray voltage was set to 1,500 V with a capillary temperature of 200°C. Full MS scans (m/z 500–850 Da) were obtained with automatic gain control target of 1 × 10^6^ ions and maximal injection time of 50 ms with lock mass m/z 529,46262. Data evaluation was performed using MassMap^®^ (MassMap, Germany).

### Blue native (BN)–PAGE and activity staining

Mitochondrial membranes were solubilized in 1% digitonin, or 0.4% dodecylmaltoside (DDM) in solubilization buffer (20 mM Tris–HCl, pH 7.4, 0.1 mM EDTA, 50 mM NaCl, 10% (w/v) glycerol, 1 mM PMSF) for 30 min at 4°C. Non‐solubilized material was removed by centrifugation for 15 min at 20,000 × *g*, 4°C, and 10× loading dye (100 mM Bis–Tris pH 7.0, 5% Coomassie brilliant blue G‐250, 500 mM ε‐amino n‐caproic acid) was added to the supernatant. Samples were separated on a 4–14% polyacrylamide gradient gel as described (Dekker *et al*, 1996). For higher resolution of supercomplexes, 3–8% polyacrylamide gels were used. Activity staining of native respiratory chain complexes separated on polyacrylamide gels was performed as described previously (Wittig *et al*, 2007). Complex I activity was visualized in staining solution containing 2.5 mg/ml nitrotetrazolium blue (NTB), 0.1 mg/ml NADH, and 5 mM Tris, pH 7.4 at 37°C until staining became visible. The staining solution for complex II contained 20 mM sodium succinate, 0.2 mM phenazine methosulfate, and 2.5 mg/ml of NTB in 5 mM Tris–HCl, pH 7.4. For complex IV staining, cytochrome c was first reduced with 15 mg/ml Na‐dithionite and subsequently added to a concentration of 1 mg/ml to the staining solution (0.5 mg/ml diaminobenzidine in 50 mM KPi, pH 7.4). Complex V staining was performed in 35 mM Tris–HCl pH 8.3, 270 mM glycine, 14 mM MgSO_4_ 0.2% Pb(NO_3_)_2_, and 8 mM ATP.

### Determination of respiratory capacity

Oxygen consumption rate (OCR) of 20,000 cardiomyocytes was measured in Seahorse XF media supplemented with 1 mM pyruvate and 4.5 g/l glucose with a XF96 Extracellular Flux Analyzer (Seahorse Bioscience, Billerica, MA, USA). Measurements were performed at basal levels, after the administration of 3 μM oligomycin, 1 μM FCCP, and 2 μM rotenone plus 1 μM antimycin A. Measurements were carried out in triplicate. Isolated cardiac, liver, or kidney mitochondria were loaded at a density of 5 μg/well into the XF 96‐well cell culture microplate by centrifugation at 20,000 × *g* for 20 min. Baseline respiration was measured at 37°C in MAS buffer (70 mM sucrose, 220 mM mannitol, 2 mM HEPES, 10 mM KH_2_PO_4_, 5 mM MgCl_2_, 1 mM EGTA, 0.2% BSA). Periodic measurements of oxygen consumption were performed after the administration of 10 mM succinate 4 mM ADP, 2 μM rotenone or 2 mM malate, 10 mM pyruvate, or 0.2 mM TMPD, 10 mM ascorbate, 4 μM antimycin A. Final measurements were performed after the administration of 2 μM antimycin A and 2 μM rotenone. Measurements were performed in eight technical replicates and repeated with the mitochondria from different mice in independent experiments.

### Quantitative PCR

RNA was isolated from the tissue samples using the SV Total RNA isolation kit (Promega) or TRIzol reagent (Invitrogen), and contaminating DNA was removed using DNase I (Thermo Scientific) according to the manufacturer's protocol. The M‐MuLV Reverse Transcriptase (Thermo Scientific) or Transcriptor High Fidelity cDNA kit (Roche) was used to reverse‐transcribe RNA into cDNA using random hexameric primers. Quantitative real‐time (RT)‐PCR was performed in triplicate using GoTaq polymerase (Promega) or SensiMix SYBR Low‐Rox One Step kit for analysis in Stratagene Mx3005P cycler. Mouse *TAZ* (Acehan *et al*, 2011) and mouse *SDHA* (5'‐ACACAGACCTGGTGGAGACC‐3′; 5'‐GGATGGGCTTGGAGTAATCA‐3′) mRNA levels were compared to β‐actin (5′‐TGTTACCAACTGGGACGACA‐3′; 5′‐GGGGTGTTGAAGGTCTCAAA‐3′). Mouse Cox1 (5'‐ATCCCTTGACATCGTGCTTC‐3′; 5'‐AAGTGGGCTTTTGCTCATGT‐3′) and Atp66 (5'‐CCTTCCACAAGGAACTCCAA‐3′; 5'‐GGTAGCTGTTGGTGGGCTAA‐3′) mRNA levels were compared to 16S rRNA (5'‐CCAATTAAGAAAGCGTTCAAG‐3′; 5'‐CCATCCAATCGGTAGTAGCG‐3′). Human *SDHA* (5'‐ACACGGACCTGGTGGAGACC‐3′; 5'‐GGATGGGCTTGGAGTAATCG‐3′) gene expression was compared to L28 (5'‐GCAATTCCTTCCGCTACAAC‐3′; 5'‐TGTTCTTGCGGATCATGTGT‐3′). Further primer sequences are available upon request.

### Measurement of enzymatic activities

Malate dehydrogenase activity was analyzed with Triton X‐100‐lysed mitochondria in an assay buffer containing 100 mM KPi, 0.1 mM NADH, and 0.2 mM oxaloacetate as a substrate, and the NADH oxidation was determined by spectrophotometrical measurement at 340 nm. For analysis of the succinate dehydrogenase, the mitochondria were disrupted by repeated freezing and thawing and then resuspended in hypotonic buffer (5 mM MgCl_2_, 25 mM KPi, pH 7.2). The activity was measured in assay buffer (50 mM KPi, 10 mM sodium succinate, 1 mM KCN, 10 μM antimycin A, 2.5 μM rotenone, pH 7.4) by the addition of 2.5 μM the ubiquinone analogue CoQ_1_ and following its reduction at an absorbance of 280 nm. For the analysis of cytochrome c oxidase activity, cytochrome c was reduced by dithionite. Reduced cytochrome c was added 1:50 to assay buffer (40 mM KPi, pH 7.5), and the reaction was started by the addition of Triton X‐100‐lysed mitochondria. The oxidation of cytochrome c was followed at 550 nm. NADH‐cytochrome c reductase activity was assessed after mitochondria were added to 0.02% (w/v) oxidized cytochrome c in assay buffer (40 mM KPi, 0.5 mM NADH, and 0.1 mM KCN, pH 7.5) and the change of absorbance at 550 nm during reduction of cytochrome c was monitored (Tzagoloff *et al*, 1975). Measurements were performed in at least three technical replicates and confirmed in independent experiments with different animals, as indicated in the figure legends.

### Miscellaneous

Isolation of mitochondria from the cardiac, kidney, and liver tissues was performed as described in Lazarou *et al* (2009). The tissue was first mechanically disrupted and then homogenized in a potter in homogenization buffer (20 mM HEPES, 220 mM mannitol, 70 mM sucrose, 1 mM EDTA, and 0.5 mM PMSF, pH 7.6). After two clarifying spins at 400 × *g* for 10 min at 4°C, the mitochondria were harvested from the supernatant by centrifugation at 11,000 × *g* for 5 min at 4°C. The generation of reactive oxygen species (ROS) of isolated mitochondria from three animals per genotype was monitored using H2DCFDA (Invitrogen). 10 μM H2DCFDA in assay buffer (20 mM Tris–HCl, 150 mM NaCl, 1% Triton X‐100, pH 7.4) was used to determine the change in fluorescence at an excitation wavelength of 498 nm and an emission wavelength of 525 nm using a fluorescence spectrophotometer (Hitachi F‐7000) for 10 min. The measurement was performed in triplicate, and the standard error was calculated. For SDS‐PAGE electrophoresis, standard techniques were used. Proteins were bound to polyvinylidene fluoride (PVDF) membranes by Western blotting and probed using primary antibodies raised in rabbit and secondary antibodies coupled to horseradish peroxidase. Commercially available antibodies used in this study are SDHA, Cell Signaling Technologies, and SDHB, Abcam. Signals were detected using the ECL detection system (GE Healthcare) and X‐ray films.

### Statistical analyses

For quantitation of sarcomere organization and quantitation of ROS production, significance was assessed using Student's *t*‐test.

### Study approval

All animal experiments were approved by the Landesamt für Verbraucherschutz und Lebensmittelsicherheit, Niedersachsen, Germany (AZ: 33.9‐42502‐04‐11/0440). The ethical committee of the University Medical Center, Göttingen, Germany, has approved the study protocol for the use of human material. Informed consent was obtained from all patients for being included in the study.

## Author contributions

JD, IFC, SG, RR, KW, SC, AC, KT, and MB performed the experiments; JD, IFC, KT, BB, FMV, RJAW, GH, KG, and PR planed the project and analyzed the data; JD, KG, and PR wrote the manuscript.

## Conflict of interest

The authors declare that they have no conflict of interest.

**The paper explained**

**Problem**

Barth syndrome (BTHS) is an inherited disease, associated with the development of cardiomyopathy. It is caused by an enzymatic defect in the synthesis of the mitochondrial membrane lipid cardiolipin, which is essential for the function of this organelle. As the generation of energy by mitochondrial oxidative phosphorylation is a common principle for all cell types, it remains unresolved why the absence of cardiolipin maturation in Barth syndrome patients predominantly affects the heart.

**Results**

Using a BTHS mouse model and patient cell‐derived cardiomyocytes, we find a tissue‐specific reorganization of the respiratory chain supercomplexes, which are required for oxidative phosphorylation. Most importantly, our data reveal a cardiac tissue‐specific loss of the tricarboxylic acid cycle enzyme succinate dehydrogenase (SDH) in BTHS. These alterations result in a dramatic loss in enzymatic functions and an increase in pathological relevant reactive oxygen species.

**Impact**

Here, we identify a molecular basis of a tissue‐specific loss of mitochondrial function in Barth syndrome.

## For more information

Please visit the website of the Barth Syndrome Foundation: https://www.barthsyndrome.org/

## Supporting information



Expanded View Figures PDF

Video EV1

Video EV2

Video EV3

Video EV4

Review Process File
