# Supplementary material for: Cardiac‐specific succinate dehydrogenase deficiency in Barth syndrome
Source: EMBO Mol Med. 2015 Dec 23;8(2):139–54. doi: 10.15252/emmm.201505644 (PMC4734842; doi:10.15252/emmm.201505644)
Supplement: Supplementary file 1 — Expanded View Figures PDF [file EMMM-8-139-s001.pdf]

Expanded View Figures

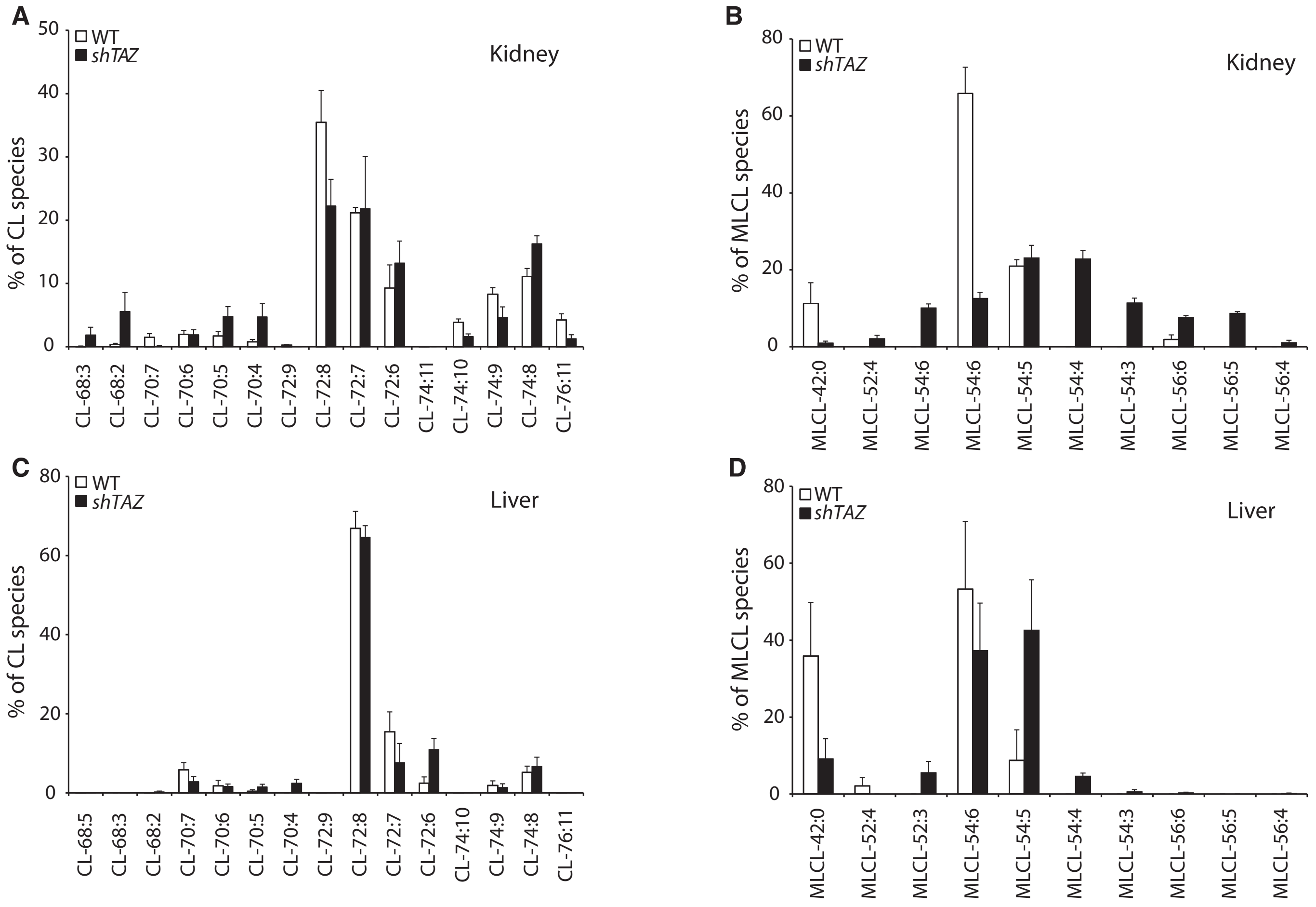

Figure EV1. Cardiolipin and monolysocardiolipin profiles in kidney and liver.

A–D Lipid species profiles of cardiolipin (A, C) and monolysocardiolipin (B, D) in the mitochondria isolated from indicated tissues analyzed by mass spectrometry. Mean of $n = 4$ per genotype, ± SEM.

© 2015 The Authors

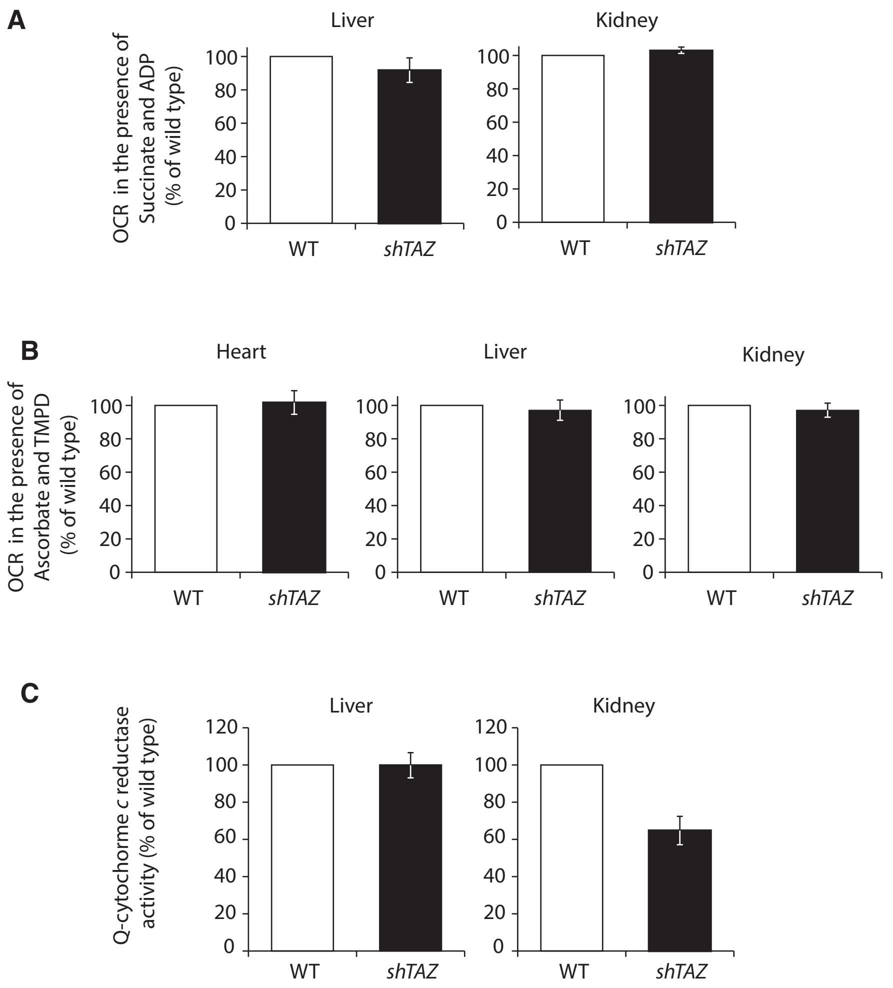

Figure EV2. Respiration is not affected in kidney and liver.

A Measurement of the oxygen consumption rate (OCR) of isolated mitochondria after the administration of succinate and ADP as substrate. Mitochondria from the liver (left) and kidney (right) were measured from 3 different animals per genotype, mean ± SEM.

B OCRs of mitochondria after the administration of TMPD and ascorbate, isolated from the heart (left; *n* = 8 per genotype), liver (central; *n* = 3 per genotype), and kidney (right; *n* = 3 per genotype); data represented in mean, ± SEM.

C Enzymatic activity of complex III in the liver (left; *n* = 3 per genotype) and kidney (right; *n* = 2 per genotype) normalized to MDH activity, in percent with control set to 100% (mean ± SEM). Complex III activity was measured spectrophotometrically as an increase in reduced cytochrome c over time.

 © 2015 The Authors
