# Supplementary material for: Cardiac‐specific succinate dehydrogenase deficiency in Barth syndrome
Source: EMBO Mol Med. 2015 Dec 23;8(2):139–54. doi: 10.15252/emmm.201505644 (PMC4734842; doi:10.15252/emmm.201505644)
Supplement: Supplementary file 2 — Video EV1 [file EMMM-8-139-s002.zip › EV Video 1 legend.rtf]

Movie EV1. Control iPSCs differentiated intocardiomyocytes that beat spontaneously at day 10.
