# Supplementary material for: Cardiac‐specific succinate dehydrogenase deficiency in Barth syndrome
Source: EMBO Mol Med. 2015 Dec 23;8(2):139–54. doi: 10.15252/emmm.201505644 (PMC4734842; doi:10.15252/emmm.201505644)
Supplement: Supplementary file 5 — Video EV4 [file EMMM-8-139-s005.zip › Video EV4.rtf]

Movie EV4. BTHS cardiomyocyte monolayers aftermetabolic selection and cultured until day 60.
