# Supplementary material for: Cardiac‐specific succinate dehydrogenase deficiency in Barth syndrome
Source: EMBO Mol Med. 2015 Dec 23;8(2):139–54. doi: 10.15252/emmm.201505644 (PMC4734842; doi:10.15252/emmm.201505644)
Supplement: Supplementary file 6 — Review Process File [file EMMM-8-139-s006.pdf]

EMBO Molecular Medicine

Cardiac specific Succinate Dehydrogenase Deficiency in Barth Syndrome

Jan Dudek, I-Fen Cheng, Arpita Chowdhury, Katharina Wozny, Martina Balleininger, Robert Reinhold, Silke Grunau, Sylvie Callegari, Karl Toischer, Ronald J. A. Wanders, Gerd Hasenfuβ, Britta Brügger, Kaomei Guan and Peter Rehling

Corresponding author: Peter Rehling, Göttingen, University

Review timeline:

	Submission date:	14 July 2015
	Accepted:	27 November 2015

Editor: Roberto Buccione

No Peer Review Process File is available with this article, as the authors have chosen not to make the review process public in this case.

© EMBO
